# Meta-GGA Density Functional Calculations on Atoms with Spherically Symmetric Densities in the Finite Element Formalism

**DOI:** 10.1021/acs.jctc.3c00183

**Published:** 2023-04-21

**Authors:** Susi Lehtola

**Affiliations:** † Molecular Sciences Software Institute, Blacksburg, Virginia 24061, United States; ‡ Department of Chemistry, University of Helsinki, P.O. Box 55, FI-00014 Helsinki, Finland

## Abstract

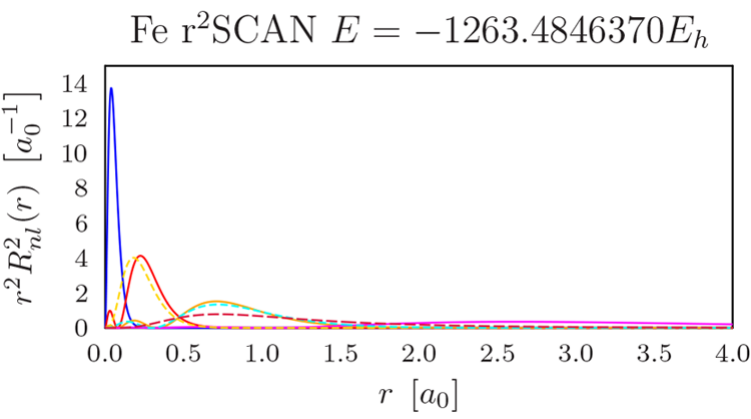

Density functional calculations on atoms are often used for determining accurate initial guesses as well as generating various types of pseudopotential approximations and efficient atomic-orbital basis sets for polyatomic calculations. To reach the best accuracy for these purposes, the atomic calculations should employ the same density functional as the polyatomic calculation. Atomic density functional calculations are typically carried out employing spherically symmetric densities, corresponding to the use of fractional orbital occupations. We have described their implementation for density functional approximations (DFAs) belonging to the local density approximation (LDA) and generalized gradient approximation (GGA) levels of theory as well as Hartree–Fock (HF) and range-separated exact exchange [Lehtola, S. *Phys. Rev. A***2020**, *101*, 012516]. In this work, we describe the extension to meta-GGA functionals using the generalized Kohn–Sham scheme, in which the energy is minimized with respect to the orbitals, which in turn are expanded in the finite element formalism with high-order numerical basis functions. Furnished with the new implementation, we continue our recent work on the numerical well-behavedness of recent meta-GGA functionals [Lehtola, S.; Marques, M. A. L. *J. Chem. Phys.***2022**, *157*, 174114]. We pursue complete basis set (CBS) limit energies for recent density functionals and find many to be ill-behaved for the Li and Na atoms. We report basis set truncation errors (BSTEs) of some commonly used Gaussian basis sets for these density functionals and find the BSTEs to be strongly functional dependent. We also discuss the importance of density thresholding in DFAs and find that all of the functionals studied in this work yield total energies converged to 0.1 *μE*_*h*_ when densities smaller than 10^–11^*a*_0_^–3^ are screened out.

## 1 Introduction

Atoms are interesting for fundamental quantum chemistry, as they form the simplest bound many-electron systems. The key aspect of the electronic structure of atoms is their shell structure, which arises from the significant amount of symmetry inherent in the Coulomb problem. Importantly for chemistry, the shell structure of atoms is preserved to a large extent also in polyatomic systems, because the nuclear Coulomb potential dominates close to the nucleus, *V*(*r*) = −*Z*/*r* → – *∞* when *r* → 0, and thus the innermost electronic orbitals turn out to be insensitive to changes in the chemical environment. This feature arguably makes atomic calculations the keystone of electronic structure calculations: the near-constant nature of the shell structure is the assumption made in most computational approaches in the electronic structure theory of polyatomic systems, as we will discuss in the following.

Quantum chemical calculations on polyatomic systems invariably start from the solution of a self-consistent field (SCF) problem.^1^ The iterative solution of the SCF problem requires an initial guess for the electron density, or the electronic orbitals. The best initial guesses are those that correctly reproduce the shell structure of atoms;^2^ good alternatives include the superposition of atomic densities^3,4^ (SAD) guess, the superposition of atomic potentials^2,5^ (SAP) guess, as well as a parameter-free extended Hückel guess^6^ that similarly can also be derived from atomic calculations.^2,7^

Also the available numerical approaches used to carry out the polyatomic calculation are tightly connected to atomic electronic structure. The dominant basis set for electronic structure calculations in the solid state is plane waves; however, such calculations invariably employ pseudopotentials^8^ or the projector augmented wave (PAW) method^9^ that eliminate the need for an explicit description for the chemically inactive core electrons. Pseudopotentials and PAW setups are again derived from atomic calculations. All-electron plane-wave calculations have only extremely recently been shown to be feasible through the use of a regularized nuclear Coulomb potential,^10,11^ but such calculations will likely be reserved for benchmark purposes due to their high computational cost.

In contrast, molecular quantum chemical calculations almost invariably employ the linear combination of atomic orbitals (LCAO) approach, in which the *i*th molecular orbital of spin σ, ψ_*iσ*_(***r***), is expressed as an expansion
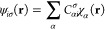
1of atomic orbitals (AOs) centered at ***R***_**α**_
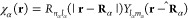
2which are composed of radial functions *R*_*nl*_ labeled by the primary quantum number *n* and angular quantum number *l*, combined with spherical harmonics *Y*_*lm*_ from *m* = −*l* to *m* = *l* in either the real or complex form, *C*_*αi*_^σ^ being the corresponding expansion coefficients. Two major advantages of LCAO calculations are that (i) all electrons can be explicitly modeled, facilitating access to electronic core properties, for instance, and that (ii) the basis set truncation errors turn out to be systematic in many cases and cancel out in chemically relevant energy differences, affording near-quantitative accuracy with basis sets of modest size.^12^

Despite their name, the (radial) AOs used in the LCAO expansion do not have to represent actual atomic orbitals, that is, physical one-particle states of an actual atoms. Instead, several types of radial functions such as Gaussian-type orbitals (GTOs) and Slater-type orbitals (STOs) may be and are commonly used in eq 2; see ref (12) for discussion. However, actual atomic orbitals are in principle the best option. These AOs can be solved by a fully numerical approach,^12^ yielding so-called numerical atomic orbitals (NAOs). NAOs are an especially powerful basis set for electronic structure calculations: the minimal NAO basis is already exact for noninteracting atoms in SCF calculations.^12,13^ Thanks to this exactness, the issues with basis set superposition error^14^ (BSSE) that complicate the determination of reliable molecular geometries in calculations with GTOs or STOs are less of an issue in calculations employing NAOs, as “borrowing” basis functions from other atoms does not lead to improvement of atomic energies. Combined with carefully formed basis sets,^15^ NAO methods have been shown to afford an excellent level of accuracy compared to fully numerical calculations.^16^ A number of programs relying on NAOs in either all-electron or pseudopotential form have been published and are in active use by the community.^15,17−22^

Because the chemical bonding situation of an atom—and the related deformation of the atom’s electron density—is not known *a priori* in a molecule, atomic starting densities, starting potentials, pseudopotentials and NAOs are typically computed using spherically symmetric densities, achieved by fractional occupations of the atomic orbitals, as this ensures that all bonding situations are described equally, on the same footing. While such an approximation may sound coarse compared to the behavior of the real atom, the approximation does yield a correct shell structure, and thus offers a simple and sensible starting point for more sophisticated calculations, such as embedding the atom in a polyatomic calculation.

Still, to reach their best accuracy, atomic starting densities, starting potentials, pseudopotentials and NAOs should be determined for the same exchange-correlation functional that is employed in the polyatomic calculation. Because different functionals lead to different orbitals, the use of inconsistent NAOs may lead to the resurrection of issues with BSSE, for instance, as the sought-for exactness property is not satisfied in such a case.

However, meta-GGA functionals are not yet supported by many atomic solvers, especially when exact exchange is also included in the functional.^12^ Although some programs have already been extended for meta-GGA functionals,^23−25^ the approaches are often not fully self-consistent. For example, the work of Sun et al.^23^ appears to have used the same PAWs for all functionals, while the recent work of Doumont et al.^26^ is likewise unable to describe core electrons and generate atomic basis functions self-consistently with meta-GGA functionals, being limited to the GGA level for full self-consistency. Many other programs still lack support for fully self-consistent meta-GGAs. We will show in this work that the self-consistent implementation of meta-GGAs for atoms with spherical symmetry is not much more demanding than that of GGA functionals.

A major motivation of this work are repeated queries for reliable atomic reference data from developers of other fully numerical approaches.^27−29^ Implementing any new computational method or algorithm requires being able to test whether the new implementation is correct, and the verification of any new atomic implementation for initial guesses, pseudopotentials or NAOs thereby requires access to reliable, high-quality reference data. Although the National Institute of Standards and Technology (NIST) hosts an atomic structure database,^30^ its content is limited to calculations performed with the local density approximation (LDA).^31,32^ While thorough data sets on some LDA and generalized gradient approximation (GGA) functionals can be found in the literature,^33,34^ sub-*μE*_*h*_ accurate reference energies for atoms with fractional occupations and meta-GGA functionals have not been published to this author’s best knowledge. A key goal of this work is to provide such highly reliable reference values for use in verifying other implementations.

A further motivation of this work is the need to characterize and study the numerical behavior of meta-GGA functionals. The present author is a long-time developer of the Libxc library of density functionals,^35^ which is used at present by some 40 electronic structure programs. We have recently thoroughly examined the numerical behavior of all the density functionals in Libxc at fixed atomic electron densities.^36^ However, as discussed in ref (36), the ultimate test for the numerical stability of density functionals is the determination of complete basis set (CBS) limit energies in fully numerical calculations, as this requires accurate evaluation of the total energy in a sequence of numerical basis sets of increasing size. Fully numerical calculations are a demanding test of the well-behavedness of density functional approximations (DFAs). The ability to run fully numerical calculations with various types of DFAs is a great boon for the development of novel DFAs, as well as for their reliable implementation in Libxc.

For all of the above reasons, it would be appealing to be able to run self-consistent calculations with meta-GGA functionals quickly and reliably in extended basis sets. The finite element method (FEM) offers an attractive solution for determining reliable NAOs and total energies. FEM affords a variational approach to the CBS limit in fully numerical calculations,^12,37^ and atomic Hartree–Fock ground-state energies converge extremely rapidly to the CBS limit with respect to the size of the radial basis set, when high-order numerical basis sets are used.^37,38^

We have previously published a general atomic solver in HelFEM^34,37,44^ that is able to handle meta-GGA functionals including global hybrids with modern finite element approaches and verified it against established Gaussian-basis approaches in ref (37). We have also recently examined the role of the finite element shape functions in meta-GGA calculations, and found that the requirements for the numerical basis set are similar for HF as well as for density functional calculations with LDA, GGA and meta-GGA functionals, and that Lagrange and Hermite interpolating polynomials can either be used to pursue the CBS limit for τ-dependent meta-GGA functionals.^38^

However, the solver described in ref (37) targets general wave functions, which may exhibit symmetry breaking, while most NAO approaches assume spherically symmetric orbitals with fractional occupations. We have recently discussed the extension of the FEM approach to the case of spherical symmetry with LDA and GGA orbitals as well as range-separated hybrids in ref (34), and reported nonrelativistic Hartree–Fock ground states for spin-unrestricted and spin-restricted calculations for H–Og (1 ≤ *Z* ≤ 118) in refs (5) and (39), respectively. The extension of the approach to meta-GGA functionals is described in this work.

The layout of this work is the following. Next, in section 2, we will outline the key pieces of FEM and present the optimal formalism for meta-GGA functionals with fractional occupations, reducing the problem into separate radial subproblems for each angular momentum *l*. Computational details including the studied selection of various LDA, GGA, and meta-GGA functionals are presented in section 3. Results of these functionals on the closed-shell and half-closed-shell atoms from H to Ar are presented in section 4. We will demonstrate that sub-*μE*_*h*_ accurate total energies can be routinely determined with the new code for various well-behaved meta-GGA functionals, and that this allows the accurate determination of the truncation errors of various Gaussian basis sets. We will also show that taking full use of the symmetry inherent in the problem yields significant speedups in the calculations, enabling calculations to be performed at the CBS limit in a matter of seconds on commodity hardware. Finally, we will examine the density thresholds employed in the various density functionals considered in this work. The article concludes in a summary and brief discussion in section 5. Atomic units are used throughout, unless specified otherwise.

## 2 Theory

In this section, we will give a brief overview of the theory necessary for implementing meta-GGAs with fractional occupations in nonrelativistic atomic calculations. We assume that the spin-σ orbitals are of the form
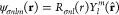
3where *R*_*σnl*_(*r*) are the spin-σ radial functions for primary quantum number *n* and angular quantum number *l*, and *Y*_*l*_^*m*^ are complex-valued spherical harmonics. When each such spin–orbital is occupied by 0 ≤ *f*_*σnlm*_ ≤ 1 electrons, the spin-σ electron density comes out as
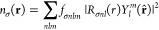
4

To achieve a spherically symmetric spin-σ electron density *n*_σ_(*r*) that only depends on the distance to the nucleus
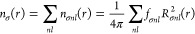
5one divides the total spin-σ occupation of shell *nl* evenly among the 2*l* + 1 magnetic sublevels as *f*_*σnlm*_ = *f*_*σnl*_/(2*l* + 1), as the Unsöld theorem^40^
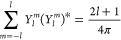
6leads to reduction of eq 4 to the radial-only form of eq 5.

The radial orbitals are expanded in a numerical basis set χ_μ_(*r*) as
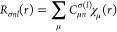
7and the energy is minimized in terms of the orbital coefficients *C*_*μn*_^σ(*l*)^. For brevity, we assume familiarity with the implementation of LDAs and GGAs in a finite basis set approach;^1^ a detailed description of the procedure for atomic finite element calculations can be found in refs (34) and (37).

Substituting eq 7 into eq 5 leads to the compact expression
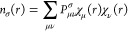
8where the density matrix
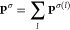
9is a sum of density matrices arising from individual angular momenta *l*
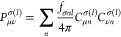
10This mathematical structure was used in ref (34) to formulate an approach for LDA, GGA, and hybrid functionals (including Hartree–Fock theory) that reduces to solving a set of coupled radial eigenvalue equations, leading to significant savings in computational and storage requirements for the wave function. In the following, we extend this approach to functionals that depend on the spin-σ local kinetic energy density τ_σ_ and/or the density Laplacian ∇^2^*n*_σ_ as
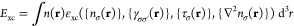
11where ϵ_xc_ is the energy density per particle that defines the used DFA and γ_*σσ*′_ = ∇*n*_σ_ ·∇*n*_σ′_ is the reduced gradient. Note that DFAs (eq 11) are often also written in terms of the energy density *f*_xc_ = *nϵ*_xc_ as
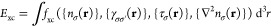
12

### 2.1 Kinetic Energy Density Dependent Functionals

The positive-definite kinetic energy density τ_σ_, which is the most popular ingredient for meta-GGAs, is given by
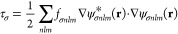
13
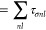
14

Following Sala et al.^41^ (see also refs (42) and (43)), τ_*σnl*_ can be rewritten as
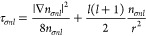
15where eq 5 gives
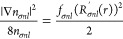
16and
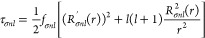
17

Substituting eq 7 into eq 17 leads to our final expression
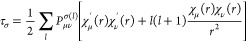
18Eq 18 is our first result: the local kinetic energy density can be rewritten as a sum of contributions from various angular momenta, which can be written solely in terms of radial density matrices. Furthermore, since we use the same radial basis set for all angular momenta,^37^eq 18 can be evaluated faster as
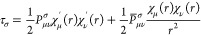
19where ***P***^σ^ was defined in eq 9 and we have introduced an angular-weighted density matrix
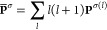
20

The final step needed for a SCF algorithm is the minimization of the total energy by variation of the orbital coefficients.^1^ As all the DFA ingredients are now seen to be spherically symmetric, the density functional contribution to the energy from eq 12 simplifies to
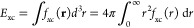
21

In the LCAO approach, varying the total energy *E* with respect to the orbital coefficients leads to the Roothaan equation ***F***^σ^***C***^σ^ = ***SC***^σ^***E***^σ^,^1^ where *F*_*μν*_^σ^ = ∂*E*/∂*P*_*μν*_^σ^ is the spin-σ Fock matrix, ***C***^σ^ and ***E***^σ^ are the orbital coefficients and the corresponding diagonal matrix of orbital energies, respectively, and ***S*** is the overlap matrix with elements *S*_*μν*_ = ⟨χ_μ_|χ_ν_⟩.^1^

The use of spherical symmetry leads to the Roothaan equation splitting into radial subproblems ***F***^σ(*l*)^***C***^σ(*l*)^ = ***S***^(*l*)^***C***^σ(*l*)^***E***^σ(*l*)^ for every angular momentum *l*, where the radial Fock matrix is
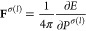
22and the radial overlap matrix is
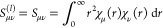
23

The LDA and GGA type contributions to the radial Fock matrix are independent of the angular momentum^34^
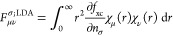
24
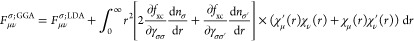
25while the kinetic energy as well as exact exchange contributions to the Fock matrix are *l* dependent with expressions given in ref (34). The τ dependence of meta-GGAs similarly leads to an *l* dependent Fock matrix contribution
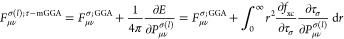
26which evaluates to
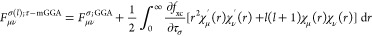
27which is the final piece of the implementation for τ-dependent functionals.

### 2.2 Density Laplacian Dependent Functionals

The Laplacian of the density, ∇^2^*n*_σ_, is straightforward to process, as the density is spherically symmetric by construction (eq 5). One must merely remember that the Laplacian has two terms in the spherical polar coordinate system:
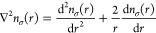
28

Substituting eq 8 into eq 28 yields
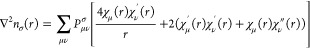
29

The Laplacian dependence leads to an additional contribution to the Fock matrix given by
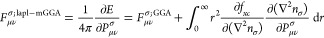
30which evaluates to the symmetric expression
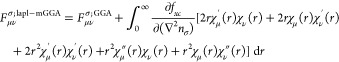
31

Note that the expression in eq 31, like the LDA and GGA contributions of eqs 24 and 25, applies to all angular momenta, at variance to the term arising from local kinetic energy density dependence in eq 27. Note also that the first two terms in the integral eq 31 can be combined with the GGA expression in eq 25, while the third term in eq 31 is of the same form as the first term in eq 27 and can be likewise evaluated together.

### 2.3 Numerical Stability Close to the Origin

The local kinetic energy τ_σ_, eq 19, seems tricky to evaluate near the origin due to the second term of eq 15, that is, *∑*_*nl*_*l*(*l* + 1)*n*_*σnl*_(*r*)/*r*^2^. However, it is easy to see that the term is regular, as only *s* orbitals have electron density at the nucleus: the *s* orbital contribution is killed as *l*(*l* + 1) = 0, while *n*_*σnl*_(*r*) → 0 when *r* → 0 for *l* > 0.

A minor complication is that although *n*_*σnl*_(*r*) ≥ 0 by definition, in practice *∑*_*nl*_*l*(*l* + 1)*n*_*σnl*_ can attain a small negative value due to finite numerical precision, which can be magnified by a large *r*^–2^ factor to generate a large negative contribution close to the nucleus. We have found that such cases only occur in the few quadrature points closest to the nucleus that carry small quadrature weights. For simplicity, we opted to stabilize this term simply by ensuring that it is non-negative by setting any negative contributions to zero.

The density Laplacian ∇^2^*n*_σ_, eq 29, has a clear singularity at the origin in its first term, where the product of the numerically stable basis functions (see eq 35 below for discussion) and their derivatives is multiplied by a singular *r*^–1^ factor. The singularity is, however, integrable, as each term becomes regular at the origin when multiplied by *r*^2^ of the quadrature weight. This also makes the Fock matrix expression of eq 31 regular, provided that *∂f*_xc_/∂(∇^2^*n*_σ_) is finite when *r* → 0.

## 3 Computational Details

### 3.1 Finite Element Calculations

All finite element calculations are performed with the HelFEM program,^34,37,44^ which employs Libxc^35^ to evaluate DFAs. The used HelFEM implementation is available in the public GitHub repository.^44^ The numerical basis functions used in this work are defined in terms of piecewise polynomial finite element shape functions *B*_μ_(*r*) as
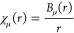
32The shape functions *B*_μ_ in eq 32 are expressed within each element *r* ∈ [*r*_*i*_, *r*_*i*+1_] in terms of a primitive coordinate *x* ∈ [−1, 1] obtained with the transformation
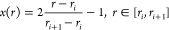
33As in our previous works on atomic calculations,^5,34,37,39^ the shape functions are chosen to be Lagrange interpolating polynomials (LIPs)
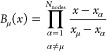
34with the nonuniform nodes {*x*_α_} chosen from the Gauss–Lobatto quadrature rule, which avoids the Runge instability^45^ and allows the use of very high-order numerical schemes. We have recently studied the use of Hermite interpolating polynomials (HIPs) instead of LIPs and found that HIPs and LIPs yield similar results with τ-dependent meta-GGA functionals.^38^

Note that although eq 32 is numerically unstable for small *r*, we have recently shown that numerically stable basis functions are afforded by Taylor expansions of eq 32 for small *r*:^38^
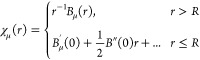
35where *R* is a switching radius that can be chosen automatically by optimal matching of the left and right-hand sides of the piecewise definition in eq 35. We employ the numerically stable form given by eq 35 with high-order Taylor series matching the polynomial order of *B*_μ_(*r*) in all calculations of this work, as discussed in ref (38).

Integrals are computed by Chebyshev quadrature with *N*_quad_ points; a rule transformed to unit weight factor is employed for this purpose, as it provides nodes and weights in easily computable analytical form.^36,37,46^ All calculations discussed in this work are converged with respect to the number of quadrature points.

The calculations employ 15-node LIPs (corresponding to 14th order polynomials), as this order was found to be sufficient for a rapid convergence of Hartree–Fock total energies in ref (37). The “exponential grid” of ref (37)
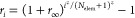
36is used in the present work with the default values *r*_*∞*_ = 40*a*_0_ and *z* = 2 for the practical infinity and the grid parameter,^37^ as these values afford excellent accuracy in Hartree–Fock calculations.^37^ We have recently shown that LDAs, GGAs, and meta-GGAs have similar grid requirements to those of Hartree–Fock.^38^

The correctness of the present implementation has been verified by direct comparison with the general implementation described in refs (34) and (37). The energy and Fock matrix reproduced by the symmetry specialized version are in exact agreement with those from the general implementation.

The SCF calculations are started from orbitals obtained from a tabulated potential from a converged LDA exchange calculation,^2,34^ and employ a combination of Pulay’s direct inversion in the iterative subspace^47,48^ (DIIS) and the augmented DIIS^49^ (ADIIS) methods for reliable SCF convergence: we typically observe convergence within a dozen of SCF iterations in the fully numerical basis set.

### 3.2 Studied Atoms

Although the presently used implementation can handle both heavy atoms and open shells (with the limitations of the presently examined nonrelativistic level of theory with a point nucleus and the use of fractional occupations),^34^ for simplicity, we will examine the H, He, Li, N, Ne, Na, P, and Ar atoms at their ground-state configurations given in Table 1. With the exception of H and He, the same atoms were also considered in our recent study on the numerical ill-behavior of density functional approximations^36^ that was motivated by this work, as many functionals were found to exhibit unsatisfactory numerical stability in SCF calculations during the preparatory phase of this manuscript. We have also recently examined the numerical well-behavedness of various recent density functionals in the H atom in ref (38).

**Table 1:** Ground-State Configurations Used in the Calculations^a^

Atom	Term symbol	Configuration	Atom	Term symbol	Configuration
H	^2^*S*	1*s*	Ne	^1^*S*	1*s*^2^2*s*^2^2*p*^6^
He	^1^*S*	1*s*^2^	Na	^2^*S*	1*s*^2^2*s*^2^2*p*^6^3*s*
Li	^2^*S*	1*s*^2^2*s*	Mg	^1^*S*	1*s*^2^2*s*^2^2*p*^6^3*s*^2^
Be	^1^*S*	1*s*^2^2*s*^2^	P	^4^*S*	1*s*^2^2*s*^2^2*p*^6^3*s*^2^3*p*^3^
N	^4^*S*	1*s*^2^2*s*^2^2*p*^3^	Ar	^1^*S*	1*s*^2^2*s*^2^2*p*^6^3*s*^2^3*p*^6^

a All calculations feature subshells that are either filled or half-filled, corresponding to an *S* state (*L* = 0) with a varying spin multiplicity shown by the left-upper hand index of the term symbol.

### 3.3 Gaussian-Basis Calculations

Importantly, the general implementation presented in ref (37) and the fractional-occupation version of this work coincide for the ground states of the atoms in Table 1 that only feature fully occupied orbitals. This also enables the direct comparison of the present results to those obtained with molecular codes employing Gaussian basis sets, for example. The correctness of the present implementation is also obvious from the excellent level of agreement between the finite element calculations and ones performed with benchmark-quality Gaussian basis sets^39^ that afford submicrohartree accuracy for light elements. The Gaussian-basis calculations in the AHGBS-9^39^ and the aug-pc-4 basis^50−55^ in fully uncontracted form (un-aug-pc-4) were performed with Erkale,^56,57^ which likewise employs Libxc for DFA evaluation. All basis functions that are unnecessary to describe the ground states of Table 1 were removed from the Gaussian basis sets. In analogy to the finite element calculations, the Gaussian-basis calculations were started from error function fitted atomic LDA exchange-only potentials.^5^

### 3.4 Studied Density Functionals

Guided by the exploratory calculations and the work presented in ref (36), the density functionals considered in this work along with their literature references are shown in Table 2.

**Table 2:** Density Functionals Studied in This Work Including the Relevant Literature References, Accompanied by the Publication Year and the Used Libxc Identifier^a^

Functional	Publication year	Libxc identifier	Type of functional
HF			GH
PW92^58−60^	1992	lda_x+lda_c_pw	LDA
PBE^61,62^	1996	gga_x_pbe+gga_c_pbe	GGA
BLYP^63,64^	1988	gga_x_b88+gga_c_lyp	GGA
B3LYP^65^	1994	hyb_gga_xc_b3lyp	GH GGA
B97^66^	1997	hyb_gga_xc_b97	GH GGA
TPSS^67^	2003	mgga_x_tpss+mgga_c_tpss	meta-GGA
revTPSS^68,69^	2009	mgga_x_revtpss+mgga_c_revtpss	meta-GGA
MS0^68,70^	2012	mgga_x_ms0+gga_c_regtpss	meta-GGA
MVS^68,71^	2015	mgga_x_mvs+gga_c_regtpss	meta-GGA
SCAN^71^	2015	mgga_x_scan+mgga_c_scan	meta-GGA
rSCAN^72^	2019	mgga_x_rscan+mgga_c_rscan	meta-GGA
r^2^SCAN^73,74^	2020	mgga_x_r2scan+mgga_c_r2scan	meta-GGA
r^2^SCAN01^25^	2022	mgga_x_r2scan01+mgga_c_r2scan01	meta-GGA
TASKCC^75,76^	2019	mgga_x_task+mgga_c_cc	meta-GGA
ωB97X-noV^77^	2014	hyb_gga_xc_wb97x_v	RSH GGA
B97M-noV^78^	2015	mgga_xc_b97m_v	meta-GGA
ωB97M-noV^79^	2016	hyb_mgga_xc_wb97m_v	RSH meta-GGA
M08-HX^80^	2008	hyb_mgga_x_m08_hx+mgga_c_m08_hx	GH meta-GGA
MN12-SX^81^	2012	hyb_mgga_x_mn12_sx+mgga_c_mn12_sx	RSH meta-GGA
MN12-L^82^	2012	mgga_x_mn12_l+mgga_c_mn12_l	meta-GGA
MN15^83^	2016	hyb_mgga_x_mn15+mgga_c_mn15	GH meta-GGA
MN15-L^84^	2016	mgga_x_mn15_l+mgga_c_mn15_l	meta-GGA
revM06^85^	2018	hyb_mgga_x_revm06+mgga_c_revm06	GH meta-GGA
revM06-L^86^	2017	mgga_x_revm06_l+mgga_c_revm06_l	meta-GGA
M06-SX^87^	2020	hyb_mgga_x_m06_sx+mgga_c_m06_sx	RSH meta-GGA
revM11^88^	2019	hyb_mgga_x_revm11+mgga_c_revm11	RSH meta-GGA

a The last column identifies the type of the functional: (semi)local LDA, GGA, or meta-GGA versus global hybrids (GHs) versus range-separated hybrids (RSHs). HF is a GH with 100% exact exchange and no semilocal energy term.

The baseline of the selection is formed by HF,^89^ the 1992 Perdew–Wang (PW92) LDA, the Perdew–Burke–Ernzerhof (PBE) and Becke–Lee–Yang–Parr (BLYP) GGAs, the B3LYP and B97 global hybrid GGAs, as well as the ωB97X-V range-separated hybrid GGA without nonlocal correlation (ωB97X-noV). This baseline composed of 7 functionals is compared to 24 meta-GGAs that consist of 15 semilocal meta-GGAs, 5 global hybrid meta-GGAs, and 4 range-separated hybrid meta-GGAs.

The meta-GGAs include the Tao–Perdew–Scuseria–Staroverov (TPSS) meta-GGA as well as its revision (revTPSS), both of whose correlation parts we have recently found to be numerically ill-behaved for alkali atoms at fixed electron density.^36^ Next, the MS0, MVS, and SCAN functionals of Perdew and co-workers were included since they have been found to exhibit successively degenerating numerical behavior.^36^

The SCAN functional has already been found to be ill-behaved in fully numerical calculations by Bartók and Yates, who suggested the rSCAN functional where the ill behavior is fixed by a well-behaved polynomial expansion.^72^ rSCAN was then later used by Furness et al.^73^ to build the r^2^SCAN functional. r^2^SCAN has showed extremely promising accuracy in applications;^90,91^ however, it has been found to be ill-behaved in fully numerical calculations by Holzwarth et al.,^25^ who proposed another modification, the r^2^SCAN01 functional. (Note that we found the whole SCAN family to be ill-behaved in ref (36).) The TASKCC functional recommended by Lebeda et al.^92^ is included as another recent meta-GGA, which appeared to be well-behaved in our recent studies.^36,38^

All the meta-GGA functionals examined in this work depend only on τ. The reason for this is that Laplacian dependence is mainly included only in older meta-GGA functionals, which are well-known to be ill-behaved. Excluding such functionals leaves only functionals that depend on τ. Deorbitalized functionals,^93^ which replace the τ dependence in recent DFAs with the density Laplacian through the use of a Laplacian-dependent kinetic energy functional are an exception to this rule; however, we have found many such functionals to be numerically ill-behaved already at fixed electron densities.^36^ Moreover, it is well-known that kinetic energy functionals have singularities near the nucleus that pose issues for numerics and the stability of SCF calculations,^94^ which are an issue even when pseudopotentials are used.^95^

The basis function requirements for τ-dependent functionals were investigated in depth in ref (38), where it was found that the LIP basis reproduces the correct CBS limit even though it does not explicitly guarantee a continuous τ by construction unlike the Hermite interpolating polynomial (HIP) basis investigated in ref (38). We tentatively attributed this success to the action of the variational theorem: as discussed in ref (37), discontinuous derivatives would lead to higher kinetic energies, which are disincentivized by the variational minimization of the total energy.

Note that calculations on Laplacian dependent functionals—which are not considered in this work—should use at least a second-order HIP basis set in order to make ∇^2^*n* well-defined everywhere. Such machinery was presented in ref (38), to which we refer for further details.

## 4 Results

### 4.1 Convergence to the Basis Set and Density Threshold Limit

We aim for total energies accurate to 0.1 *μE*_*h*_ with respect to all parameteres in the present calculations. In practice, we determine that two calculations have converged to within 0.1 *μE*_*h*_ precision if the energies *E*_1_ and *E*_2_ of the two calculations agree within 0.04 *μE*_*h*_: |*E*_1_ – *E*_2_| < 4 × 10^–8^.

We therefore carry out a large number of calculations to determine reference total energies converged with respect to all the parameters controlling the calculation: in addition to the number of radial basis functions, we also study the convergence with respect to the number of quadrature points, as well as the effect of the density screening threshold employed in the DFA implementation.

When the values of the density functional and its derivatives are evaluated on the numerical quadrature grid by Libxc,^35^ points with insignificant electron density *n*(***r***) ≤ ϵ as defined by a preset threshold ϵ > 0 are skipped, setting the energy density *f*_xc_ and all its derivatives to zero. The rationale for such thresholding is grounded on the physical observation that both factors in the energy density *f*_xc_ = *nϵ*_xc_(*n*, ...) decrease when *n* → 0 and therefore points with negligible density do not contribute meaningfully to the total energy; a practical issue is also that points with extremely small densities often yield divergent derivatives. Because of this, such thresholding is commonly used in density functional implementations.

This also extends further: typical density functional quadrature approaches discard basis functions with negligible values. For instance, the approach of Stratmann et al.^96^ discards atomic basis functions χ_α_(***r***) in the sphere around the atom where  with ; such an approach also leads to errors in the electron density, and the approach thereby relies on the small errors in the density not having a significant effect on the total energy.

A threshold ϵ = 10^–12^ has been used in previous studies with HelFEM, which is also the default in the Psi4^97^ and ORCA^98^ programs, for example. In this work, we determine the density cutoff ϵ used in the evaluation of the density functional, specified with the Libxc function xc_func_set_dens_threshold, by considering a series of calculations performed with decreasing values in ϵ = 10^–*n*^ with *n* = 8, *...*, 15. (The default value used in Libxc for most density functionals for three-dimensional systems is ϵ = 10^–15^, below which value numerical issues are often encountered due to the use of double precision arithmetic.) We deem convergence to have been reached at the largest value of ϵ for which the energy does not change any more.

We wish to underline here that the use of density thresholds in this work is not an approximation that is special to this work, as such finite thresholds are always used to ensure that the numerical implementation of any functional is stable. Instead, the density threshold should be considered part of the definition of the DFA, and a threshold that is small enough to reproduce the converged value should always be used. Unfortunately, the issue is that the values of the used thresholds are typically not discussed in articles suggesting novel DFAs. However, we do find that the studied functionals converge quickly in the density threshold.

We determine the convergence to the CBS limit by considering calculations with a sequence of increasing numerical basis sets composed of 5, 10, 15, 20, 25, 30, and 35 radial 15-node LIP elements with with 69, 139, 209, 279, 349, 419, and 489 radial basis functions, respectively. Convergence to the CBS limit is established when the difference in total energy to the calculation with the largest number of radial basis functions is below the wanted precision. The convergence with respect to the quadrature is checked by doubling the used number of quadrature points, and checking whether the total energies of the SCF calculations performed with different quadratures agree.

All fully numerical calculations failed with the MVS and SCAN functionals, and these functionals were excluded from all analyses; the ill-behavedness of SCAN was already reported by Bartók and Yates.^72^ The obtained fully numerical reference energies for the remaining functionals are shown where available in Table 4 for H, He, Li, Be, and N and in Table 5 for Ne, Na, Mg, P, and Ar. Our analysis in Tables 4 and 5 also distinguishes cases where reliable reference energies were not achieved by the present computational scheme due to (i) failures with SCF convergence, (ii) failures with achieving the CBS limit, and (iii) floating point errors in the calculation.

As expected from our previous experience, Li, Be, Na, and Mg are a challenge for many functionals due to their loosely bound outer electrons, as can be seen from the large number of failed calculations for these atoms. Although the initial guess is good, as suggested by DIIS errors in the range of 10^–2^, we observe that many of the failed calculations go wrong already in the first iteration. For instance, the revM06, revM06-L, M06-SX, MN12-L, MN12-SX, MN15, B97M-V, and ωB97M-V calculations on Li start from a sensible total energy from the initial guess, but jump up thousands of Hartrees in energy upon the diagonalization of the first Fock matrix, which suggests that there are issues in these functionals’ numerical behavior for some densities.

These observations motivate a systematical examination of the initial guess. The largest DIIS errors and largest energy changes in the first iteration that arise from the first full diagonalization of the Fock matrix are shown in Table 3. The functionals that stand out with a large DIIS error are MS0 and MVS, and all nine Minnesota functionals. The Ar atom has the largest initial DIIS error out of the studied atoms and functionals, with the exception of PW92 that encounters its largest initial DIIS error for the N atom.

**Table 3:** Largest Initial DIIS Error ϵ_DIIS_ and the Largest Energy Change in the First Iteration in the Calculations with 35 Radial Elements and a Density Threshold ϵ = 10^–12^^a^

Method	Atom	ϵ_DIIS_	Atom	Δ*E*
HF	Ar	5.4 × 10^–2^	He	–2.8 × 10^–3^
PW92	N	2.4 × 10^–3^	He	–2.2 × 10^–4^
PBE	Ar	4.9 × 10^–2^	He	–1.6 × 10^–3^
BLYP	Ar	5.4 × 10^–2^	He	–2.0 × 10^–3^
B3LYP	Ar	3.9 × 10^–2^	He	–1.9 × 10^–3^
B97	Ar	4.2 × 10^–2^	Li	7.2 × 10^0^
TPSS	Ar	5.0 × 10^–2^	He	–2.6 × 10^–3^
revTPSS	Ar	5.6 × 10^–2^	He	–2.6 × 10^–3^
MS0	Ar	2.0 × 10^–1^	He	–2.4 × 10^–3^
MVS	Ar	4.2 × 10^–1^	He	–2.4 × 10^–3^
rSCAN	Ar	5.1 × 10^–2^	He	–2.2 × 10^–3^
r^2^SCAN	Ar	5.5 × 10^–2^	He	–2.2 × 10^–3^
r^2^SCAN01	Ar	5.5 × 10^–2^	He	–2.2 × 10^–3^
TASKCC	Ar	8.4 × 10^–2^	He	–3.5 × 10^–3^
ωB97X-noV	Ar	4.8 × 10^–2^	He	–1.8 × 10^–3^
B97M-noV	Ar	7.3 × 10^–2^	Li	5.7 × 10^4^
ωB97M-noV	Ar	8.0 × 10^–2^	Li	5.7 × 10^4^
M08-HX	Ar	4.9 × 10^–1^	H	–4.3 × 10^–3^
MN12-SX	Ar	1.2 × 10^0^	Na	1.1 × 10^5^
MN12-L	Ar	5.8 × 10^–1^	Li	7.6 × 10^4^
MN15	Ar	1.5 × 10^0^	Li	1.1 × 10^5^
MN15-L	Ar	1.5 × 10^0^	H	–4.0 × 10^–3^
revM06	Ar	1.5 × 10^–1^	Li	3.1 × 10^3^
revM06-L	Ar	2.5 × 10^–1^	Na	5.4 × 10^3^
M06-SX	Ar	1.5 × 10^–1^	Li	3.1 × 10^3^
revM11	Ar	2.6 × 10^–1^	He	–2.7 × 10^–3^

a The corresponding atoms are also shown.

Interestingly, the initial DIIS error does not appear to correlate strongly with the initial change in energy. Only B97, the Berkeley meta-GGA functionals B97M-noV and ωB97M-noV, and six out of nine Minnesota functionals show an increase in energy upon the first diagonalization; however, these increases are alarmingly large. We note again that the largest stability problems appear to be encountered with the Li and Na atoms, as can be seen from Table 3.

Our baseline of HF, PW92, PBE, BLYP, B3LYP, and B97 converge without issues, with the exceptions of Li and N with B97. The B97 functional appears to be less smooth than the other baseline functionals, as evidenced by its need for more radial elements to reach the same convergence.

The TPSS, revTPSS and TASKCC functionals are well-behaved, easily converging to the CBS limit for all atoms with the exception of Li, which required surprisingly many elements.

The MS0 functional is ill-behaved, failing to reach the CBS limit for Li, Be, Na, Mg, and P even with the extended numerical basis sets considered in this work.

The rSCAN functional is well-behaved, other than failing to reach the CBS limit for Li. In partial agreement with Holzwarth et al.,^25^ we find that the r^2^SCAN01 functional is better-behaved than r^2^SCAN, as calculations failed for Li and Na for the latter functional. However, we do not find evidence that r^2^SCAN01 is otherwise smoother, as the functional still takes more radial elements to converge than the well-behaved TPSS, revTPSS or TASKCC functionals, and as r^2^SCAN01 required more radial elements to converge P than r^2^SCAN did.

The ωB97X-noV range-separated hybrid GGA is well-behaved and converges easily for all studied systems. In contrast, the B97M-noV and ωB97M-noV meta-GGAs fail to converge for Li, N, Na, and P. All of the failed calculations are characterized by large jumps in energy in the first iteration, as discussed above for several functionals with Li and Na.

The nine Minnesota functionals appear well-behaved for H, He, N, Ne, and Ar, as all nine functionals reach converged CBS limit energies. The M08-HX functional, however, requires many more radial elements than the other functionals, which can be explained by our recent observation in ref (38) of sharp nonphysical behavior in the functional. No Minnesota functional is successful for Li, while only revM11 is able to reach a converged CBS limit for Na. Only MN15, revM06, revM06-L, and M06-SX reach CBS limits for Be. For Mg, the CBS limit is reached by MN15, MN15-L, revM06-L, and revM11. All the studied Minnesota functionals except MN12-SX reach a CBS limit for P.

The converged density thresholds are shown in Table 6; the systems that failed to converge in Table 4 were excluded in this analysis. All functionals reach total energies converged to 0.1 *μE*_*h*_ with a density threshold of ϵ = 10^–11^ or larger, confirming the general reliability of the universally used screening approach, and confirming the reliability of the results previously obtained with the default threshold ϵ = 10^–12^.

**Table 4:** Reference Energies in *E*_*h*_ with the Finite Element Method/Number of Radial Elements Required to Reach the Converged Energy for the H, He, Li, Be, and N Atoms^a^

Functional	H	He	Li	Be	N
HF	–0.5000000/5	–2.8616800/5	–7.4327509/5	–14.5730232/5	–54.4045483/5
PW92	–0.4787107/5	–2.8344552/5	–7.3432842/5	–14.4464735/5	–54.1343867/5
PBE	–0.4999904/5	–2.8929349/5	–7.4621804/10	–14.6299477/10	–54.5357555/5
BLYP	–0.4979143/5	–2.9070669/5	–7.4826660/5	–14.6615080/5	–54.5931773/5
B3LYP	–0.5024433/5	–2.9152187/5	–7.4929571/5	–14.6733282/5	–54.6070284/5
B97	–0.5029846/5	–2.9099945/5	NoSCF	–14.6671376/10	NoSCF
TPSS	–0.5002355/5	–2.9096639/5	–7.4891131/15	–14.6717170/10	–54.6161733/10
revTPSS	–0.5001577/5	–2.9120536/5	–7.4901709/20	–14.6725883/10	–54.5978896/10
TASKCC	–0.5001730/5	–2.9794485/5	–7.5620745/15	–14.7471258/10	–54.6252881/10
MS0	–0.5066733/5	–2.9115322/5	NoCBS	NoCBS	–54.6179098/20
rSCAN	–0.5001732/5	–2.9049561/5	NoCBS	–14.6511980/10	–54.5993803/20
r^2^SCAN	–0.5001732/5	–2.9049561/5	NoSCF	–14.6490866/15	–54.5840337/20
r^2^SCAN01	–0.5001732/5	–2.9049561/5	–7.4800036/20	–14.6496022/15	–54.5860431/20
ωB97X-noV	–0.5053272/5	–2.9127069/5	–7.4924999/5	–14.6805446/5	–54.6197329/5
B97M-noV	–0.5061077/5	–2.9367807/5	NoSCF	–14.7081142/10	NoSCF
ωB97M-noV	–0.4992064/5	–2.9080420/5	NoSCF	–14.6807256/10	NoSCF
M08-HX	–0.5039981/10	–2.9181530/20	NoSCF	NoCBS	–54.5963110/20
MN12-SX	–0.4970768/5	–2.9170941/10	NoSCF	NoSCF	–54.5845822/15
MN12-L	–0.4923232/5	–2.9156167/10	NoSCF	NoCBS	–54.5673401/10
MN15	–0.4997453/5	–2.9219234/5	NoSCF	–14.6808919/15	–54.5889705/10
MN15-L	–0.4965988/5	–2.9161651/5	NoSCF	NoCBS	–54.5963736/10
revM06	–0.4978698/5	–2.9129975/5	NoSCF	–14.6643064/15	–54.5822636/10
revM06-L	–0.5000720/5	–2.9239856/5	NoSCF	–14.6738826/20	–54.5927481/10
M06-SX	–0.4856891/5	–2.8992554/5	NoSCF	–14.6468389/10	–54.5676518/10
revM11	–0.5023467/5	–2.8975261/5	NoCBS	NoCBS	–54.5778168/10

a Calculations for which reliable reference energies marked as follows. NoSCF: the SCF procedure failed to converge. NoQuad: the numerical quadrature was not converged. NoCBS: a converged energy was not reached. NaN: a not-a-number floating point error was encountered in the calculations.

**Table 5:** Reference Energies in *E*_*h*_ with the Finite Element Method/Number of Radial Elements Required to Reach the Converged Energy for the Ne, Na, Mg, P, and Ar Atoms^a^

Functional	Ne	Na	Mg	P	Ar
HF	–128.5470981/5	–161.8589538/5	–199.6146364/5	–340.7192753/5	–526.8175128/5
PW92	–128.2299172/5	–161.4436320/5	–199.1352883/5	–340.0000523/5	–525.9397934/5
PBE	–128.8664277/5	–162.1726872/5	–199.9551151/5	–341.1156817/10	–527.3461288/5
BLYP	–128.9730149/5	–162.2927034/5	–200.0926430/5	–341.2778807/5	–527.5510394/5
B3LYP	–128.9809732/5	–162.3031506/5	–200.1035499/5	–341.2928849/5	–527.5678350/5
B97	–128.9418808/10	–162.2557399/10	–200.0507705/10	–341.2270703/10	–527.4847536/10
TPSS	–128.9811078/10	–162.2986086/10	–200.0927812/10	–341.2963243/10	–527.5694173/10
revTPSS	–128.9242010/10	–162.2273272/10	–200.0077708/10	–341.1618278/10	–527.3782603/10
TASKCC	–128.9847733/10	–162.2753046/10	–200.0383120/15	–341.1235642/10	–527.3566109/10
MS0	–128.9818356/15	NoCBS	NoCBS	NoCBS	–527.5880852/20
rSCAN	–128.9723924/10	–162.2983845/20	–200.0958519/15	–341.3288363/15	–527.6283474/15
r^2^SCAN	–128.9348395/10	NoSCF	–200.0443066/15	–341.2500476/15	–527.5177200/15
r^2^SCAN01	–128.9394874/10	–162.2600473/15	–200.0501446/15	–341.2582840/20	–527.5287026/15
ωB97X-noV	–128.9808707/5	–162.2959918/5	–200.0901465/5	–341.2765096/5	–527.5527095/10
B97M-noV	–128.9741484/10	NoSCF	–200.0825748/15	NoSCF	–527.4912402/10
ωB97M-noV	–128.9939646/10	NoSCF	–200.1215024/10	NaN	–527.6026146/10
M08-HX	–128.9488829/10	NoCBS	NoCBS	–341.2642177/15	–527.5522818/20
MN12-SX	–128.9439150/10	NoSCF	NoCBS	NoSCF	–527.5637752/15
MN12-L	–128.9511777/10	NoSCF	NoSCF	–341.2968361/15	–527.5498561/15
MN15	–128.9582835/10	NoCBS	–200.0789652/10	–341.2689683/10	–527.6036546/10
MN15-L	–128.9359083/10	NoCBS	–200.0757004/15	–341.2873822/15	–527.5886820/10
revM06	–128.9455051/10	NoSCF	NoSCF	–341.2549085/10	–527.5410881/10
revM06-L	–128.9535087/10	NoSCF	–200.0648304/15	–341.2576943/10	–527.5364675/10
M06-SX	–128.9442104/10	NoSCF	NoSCF	–341.2612515/10	–527.5470863/10
revM11	–128.9442958/10	–162.2556667/10	–200.0475869/10	–341.2534754/10	–527.5401522/10

a The notation is analogous to that in Table 4.

**Table 6:** Largest Density Threshold ϵ ∈ [10^–8^, 10^–9^, *...*, 10^–15^] That Reproduces the Reference Energies of Tables 4 and 5 to 0.1 *μE*_*h*_^a^

Functional	Threshold
PW92	10^–9^
PBE	10^–10^
BLYP	10^–11^
B3LYP	10^–11^
B97	10^–11^
TPSS	10^–10^
revTPSS	10^–10^
TASKCC	10^–10^
MS0	10^–10^
rSCAN	10^–9^
r^2^SCAN	10^–10^
r^2^SCAN01	10^–10^
ωB97X-noV	10^–10^
B97M-noV	10^–10^
ωB97M-noV	10^–10^
M08-HX	10^–10^
MN12-SX	10^–11^
MN12-L	10^–11^
MN15	10^–11^
MN15-L	10^–11^
revM06	10^–10^
revM06-L	10^–10^
M06-SX	10^–10^
revM11	10^–10^

a Calculations that failed were not included in the analysis.

### 4.2 Gaussian-Basis Truncation Errors

Furnished with the converged fully numerical reference energies, we are able to determine truncation errors of Gaussian basis sets. This part of the study is partly motivated by our recent work in ref (99), where we observed unexpectedly large basis set truncation errors for hydrogen with the M06-L,^100^ M11-L,^101^ and B97M-noV functionals. We later found M06-L and M11-L to be ill-behaved,^38^ exhibiting large oscillations in the density Laplacian ∇^2^*n* in the ground state of the hydrogen atom, which explains the large differences in energies in fully numerical and Gaussian-basis calculations.

The question of the accuracy of meta-GGA functional energies in Gaussian basis sets has not been addressed in the literature so far to the best of our knowledge. The recent study of Kraus^102^ studied basis set extrapolations with modern density functionals using HelFEM, but does not appear to comment on the functional dependence of the accuracy in total energy.

We consider the aug-pc-4 basis set^50−55^ in its fully uncontracted form (un-aug-pc-4), and our recent augmented hydrogenic Gaussian basis set (AHGBS-9).^39^ The un-aug-pc-4 basis set has been optimized for the BLYP functional,^51,54,55^ while the AHGBS-9 basis set and its polarized counterparts are constructed by considerations on one-electron ions, only.^39^ The hydrogenic basis sets of ref (39) are large even-tempered basis sets aimed for benchmark accuracy calculations on atoms and molecules. Note that even-tempered basis sets are often used for studies on basis set completeness for their favorable properties.^103−105^

The truncation errors for the un-aug-pc-4 basis set are shown in Table 7, and the errors for AHGBS-9 basis set are shown in Table 8. The Gaussian-basis energies were determined with the basis set truncation and density screening thresholds ϵ = 10^–12^ and , respectively, and a (500, 974) quadrature grid.

**Table 7:** Truncation Errors in *E*_*h*_ for the un-aug-pc-4 Gaussian Basis Set, Computed Using the Reference Energies Accurate to 10^–7^*E*_*h*_ Given in Tables 4 and 5^a^

Functional	H	He	Li	Be	N	Ne	Na	Mg	P	Ar
HF	4.1 × 10^–7^	4.8 × 10^–6^	9.4 × 10^–7^	1.5 × 10^–6^	5.3 × 10^–6^	1.8 × 10^–5^	1.2 × 10^–5^	1.3 × 10^–5^	1.6 × 10^–5^	2.3 × 10^–5^
PW92	3.2 × 10^–7^	3.9 × 10^–6^	9.4 × 10^–7^	1.5 × 10^–6^	4.8 × 10^–6^	1.6 × 10^–5^	9.9 × 10^–6^	1.1 × 10^–5^	1.1 × 10^–5^	1.8 × 10^–5^
PBE	5.3 × 10^–7^	3.2 × 10^–6^	1.1 × 10^–5^	7.3 × 10^–6^	9.4 × 10^–6^	1.3 × 10^–5^	5.9 × 10^–5^	4.8 × 10^–5^	3.1 × 10^–5^	2.6 × 10^–5^
BLYP	3.4 × 10^–7^	3.2 × 10^–6^	2.3 × 10^–6^	3.3 × 10^–6^	5.5 × 10^–6^	1.2 × 10^–5^	1.1 × 10^–5^	9.0 × 10^–6^	1.4 × 10^–5^	1.7 × 10^–5^
B3LYP	3.4 × 10^–7^	3.3 × 10^–6^	1.7 × 10^–6^	2.5 × 10^–6^	4.8 × 10^–6^	1.3 × 10^–5^	1.1 × 10^–5^	8.9 × 10^–6^	1.2 × 10^–5^	1.5 × 10^–5^
B97	2.4 × 10^–6^	3.3 × 10^–6^	N/A	1.2 × 10^–5^	N/A	2.2 × 10^–5^	1.8 × 10^–4^	4.4 × 10^–5^	1.2 × 10^–4^	8.3 × 10^–5^
TPSS	4.0 × 10^–6^	7.8 × 10^–6^	9.1 × 10^–5^	1.2 × 10^–4^	1.2 × 10^–4^	3.4 × 10^–5^	1.9 × 10^–4^	1.7 × 10^–4^	2.1 × 10^–4^	3.2 × 10^–4^
revTPSS	4.0 × 10^–6^	5.6 × 10^–6^	8.7 × 10^–5^	1.1 × 10^–4^	1.3 × 10^–4^	3.9 × 10^–5^	1.7 × 10^–4^	1.7 × 10^–4^	1.5 × 10^–4^	1.7 × 10^–4^
TASKCC	3.7 × 10^–7^	4.6 × 10^–6^	1.9 × 10^–4^	3.5 × 10^–4^	5.1 × 10^–4^	3.4 × 10^–4^	5.4 × 10^–4^	7.9 × 10^–4^	8.5 × 10^–4^	8.2 × 10^–4^
MS0	3.3 × 10^–7^	3.9 × 10^–6^	N/A	N/A	2.4 × 10^–4^	2.3 × 10^–4^	N/A	N/A	N/A	9.4 × 10^–4^
rSCAN	3.6 × 10^–7^	4.5 × 10^–6^	N/A	5.7 × 10^–5^	1.0 × 10^–4^	5.8 × 10^–5^	1.0 × 10^–4^	1.4 × 10^–4^	2.4 × 10^–4^	1.8 × 10^–4^
r^2^SCAN	3.7 × 10^–7^	4.5 × 10^–6^	N/A	6.5 × 10^–5^	1.2 × 10^–4^	8.3 × 10^–5^	N/A	1.5 × 10^–4^	2.8 × 10^–4^	2.3 × 10^–4^
r^2^SCAN01	3.6 × 10^–7^	4.5 × 10^–6^	1.0 × 10^–4^	6.2 × 10^–5^	1.2 × 10^–4^	8.2 × 10^–5^	1.6 × 10^–4^	1.5 × 10^–4^	2.8 × 10^–4^	2.3 × 10^–4^
ωB97X-noV	4.4 × 10^–7^	3.4 × 10^–6^	2.2 × 10^–6^	4.2 × 10^–6^	6.6 × 10^–6^	1.6 × 10^–5^	1.8 × 10^–5^	1.9 × 10^–5^	2.1 × 10^–5^	2.5 × 10^–5^
B97M-noV	1.5 × 10^–5^	1.9 × 10^–5^	N/A	8.4 × 10^–4^	N/A	7.6 × 10^–5^	N/A	5.5 × 10^–4^	N/A	2.9 × 10^–4^
ωB97M-noV	6.7 × 10^–6^	7.1 × 10^–6^	N/A	8.1 × 10^–5^	N/A	2.5 × 10^–5^	N/A	3.5 × 10^–4^	N/A	1.3 × 10^–4^
M08-HX	3.6 × 10^–4^	2.4 × 10^–3^	N/A	N/A	2.3 × 10^–3^	3.6 × 10^–3^	N/A	N/A	5.3 × 10^–3^	8.2 × 10^–3^
MN12-SX	1.5 × 10^–4^	7.4 × 10^–4^	N/A	N/A	2.9 × 10^–3^	2.5 × 10^–3^	N/A	N/A	N/A	5.6 × 10^–3^
MN12-L	6.8 × 10^–5^	1.5 × 10^–3^	N/A	N/A	2.5 × 10^–3^	4.3 × 10^–3^	N/A	N/A	6.5 × 10^–3^	1.1 × 10^–2^
MN15	1.7 × 10^–5^	3.4 × 10^–5^	N/A	2.7 × 10^–4^	4.1 × 10^–4^	2.4 × 10^–4^	N/A	1.4 × 10^–3^	2.0 × 10^–3^	2.1 × 10^–3^
MN15-L	1.5 × 10^–5^	4.4 × 10^–4^	N/A	N/A	1.0 × 10^–3^	9.0 × 10^–4^	N/A	1.6 × 10^–3^	2.3 × 10^–3^	2.4 × 10^–3^
revM06	9.7 × 10^–5^	2.5 × 10^–4^	N/A	3.2 × 10^–4^	3.2 × 10^–4^	4.6 × 10^–4^	N/A	N/A	4.3 × 10^–4^	5.7 × 10^–4^
revM06-L	1.6 × 10^–4^	3.5 × 10^–4^	N/A	3.6 × 10^–4^	4.9 × 10^–4^	6.8 × 10^–4^	N/A	1.2 × 10^–3^	1.1 × 10^–3^	1.3 × 10^–3^
M06-SX	6.1 × 10^–5^	1.7 × 10^–4^	N/A	1.9 × 10^–4^	1.9 × 10^–4^	3.0 × 10^–4^	N/A	N/A	2.3 × 10^–4^	4.0 × 10^–4^
revM11	6.1 × 10^–5^	3.2 × 10^–5^	N/A	N/A	3.5 × 10^–4^	2.1 × 10^–4^	2.7 × 10^–4^	4.5 × 10^–4^	8.6 × 10^–4^	8.9 × 10^–4^

a N/A: The data are not available as converged fully numerical energies could not be determined.

**Table 8:** Truncation Errors in *E*_*h*_ for the AHGBS-9 Gaussian Basis Set, Computed Using the Reference Energies Accurate to 10^–7^*E*_*h*_ Given in Tables 4 and 5^a^

Functional	H	He	Li	Be	N	Ne	Na	Mg	P	Ar
HF	∼0	∼0	∼0	∼0	1.3 × 10^–7^	3.3 × 10^–7^	4.4 × 10^–7^	5.7 × 10^–7^	1.1 × 10^–6^	2.1 × 10^–6^
PW92	∼0	∼0	4.9 × 10^–8^	5.7 × 10^–8^	1.4 × 10^–7^	3.3 × 10^–7^	4.9 × 10^–7^	6.8 × 10^–7^	1.4 × 10^–6^	2.6 × 10^–6^
PBE	∼0	∼0	1.5 × 10^–5^	4.9 × 10^–6^	2.8 × 10^–6^	1.1 × 10^–6^	1.1 × 10^–5^	4.0 × 10^–6^	1.8 × 10^–5^	2.2 × 10^–5^
BLYP	∼0	7.4 × 10^–8^	3.3 × 10^–6^	3.3 × 10^–6^	1.2 × 10^–6^	8.7 × 10^–7^	1.6 × 10^–6^	1.5 × 10^–6^	8.2 × 10^–6^	1.4 × 10^–5^
B3LYP	∼0	6.1 × 10^–8^	1.8 × 10^–6^	1.9 × 10^–6^	7.1 × 10^–7^	6.3 × 10^–7^	1.1 × 10^–6^	1.2 × 10^–6^	5.0 × 10^–6^	8.4 × 10^–6^
B97	1.1 × 10^–7^	∼0	N/A	9.5 × 10^–6^	N/A	3.0 × 10^–6^	2.3 × 10^–5^	5.8 × 10^–6^	8.5 × 10^–5^	6.3 × 10^–5^
TPSS	1.4 × 10^–7^	∼0	9.0 × 10^–5^	1.3 × 10^–4^	6.8 × 10^–5^	5.8 × 10^–6^	3.5 × 10^–5^	7.4 × 10^–5^	1.3 × 10^–4^	1.5 × 10^–4^
revTPSS	2.3 × 10^–7^	1.2 × 10^–7^	8.2 × 10^–5^	1.1 × 10^–4^	8.3 × 10^–5^	7.8 × 10^–6^	4.8 × 10^–5^	1.0 × 10^–4^	1.2 × 10^–4^	1.2 × 10^–4^
TASKCC	∼0	∼0	2.9 × 10^–4^	3.7 × 10^–4^	2.1 × 10^–4^	2.9 × 10^–5^	2.7 × 10^–4^	3.9 × 10^–4^	6.7 × 10^–4^	4.9 × 10^–4^
MS0	∼0	∼0	N/A	N/A	2.0 × 10^–4^	2.0 × 10^–4^	N/A	N/A	N/A	7.6 × 10^–4^
rSCAN	∼0	∼0	N/A	4.9 × 10^–5^	7.3 × 10^–5^	1.8 × 10^–5^	6.5 × 10^–5^	6.7 × 10^–5^	1.6 × 10^–4^	1.3 × 10^–4^
r^2^SCAN	∼0	∼0	N/A	5.4 × 10^–5^	8.5 × 10^–5^	2.7 × 10^–5^	N/A	8.6 × 10^–5^	2.1 × 10^–4^	1.8 × 10^–4^
r^2^SCAN01	∼0	∼0	1.1 × 10^–4^	5.2 × 10^–5^	8.3 × 10^–5^	2.7 × 10^–5^	1.1 × 10^–4^	8.5 × 10^–5^	2.1 × 10^–4^	1.8 × 10^–4^
ωB97X-noV	∼0	∼0	2.6 × 10^–6^	1.8 × 10^–6^	2.3 × 10^–6^	9.7 × 10^–7^	2.8 × 10^–6^	2.9 × 10^–6^	7.4 × 10^–6^	1.7 × 10^–5^
B97M-noV	5.2 × 10^–6^	1.2 × 10^–7^	N/A	8.5 × 10^–4^	N/A	2.5 × 10^–5^	N/A	2.9 × 10^–4^	N/A	2.8 × 10^–4^
ωB97M-noV	4.9 × 10^–7^	1.0 × 10^–6^	N/A	1.4 × 10^–4^	N/A	3.8 × 10^–6^	N/A	2.3 × 10^–4^	N/A	7.0 × 10^–5^
M08-HX	3.2 × 10^–5^	4.7 × 10^–4^	N/A	N/A	1.4 × 10^–3^	2.1 × 10^–3^	N/A	N/A	3.8 × 10^–3^	7.6 × 10^–3^
MN12-SX	1.6 × 10^–5^	1.0 × 10^–4^	N/A	N/A	1.3 × 10^–3^	4.8 × 10^–4^	N/A	N/A	N/A	3.7 × 10^–3^
MN12-L	1.3 × 10^–6^	1.4 × 10^–4^	N/A	N/A	1.1 × 10^–3^	9.1 × 10^–4^	N/A	N/A	3.7 × 10^–3^	7.2 × 10^–3^
MN15	9.3 × 10^–7^	6.9 × 10^–7^	N/A	2.5 × 10^–4^	1.8 × 10^–4^	3.5 × 10^–5^	N/A	4.1 × 10^–4^	1.2 × 10^–3^	1.1 × 10^–3^
MN15-L	2.3 × 10^–6^	3.3 × 10^–6^	N/A	N/A	4.5 × 10^–4^	1.2 × 10^–4^	N/A	7.5 × 10^–4^	1.9 × 10^–3^	1.5 × 10^–3^
revM06	7.1 × 10^–5^	8.3 × 10^–5^	N/A	3.1 × 10^–4^	2.2 × 10^–4^	1.6 × 10^–4^	N/A	N/A	3.8 × 10^–4^	4.5 × 10^–4^
revM06-L	3.5 × 10^–5^	1.2 × 10^–5^	N/A	4.1 × 10^–4^	1.4 × 10^–4^	8.4 × 10^–5^	N/A	4.8 × 10^–4^	7.6 × 10^–4^	8.2 × 10^–4^
M06-SX	2.0 × 10^–5^	1.0 × 10^–5^	N/A	2.0 × 10^–4^	8.0 × 10^–5^	3.4 × 10^–5^	N/A	N/A	1.7 × 10^–4^	1.8 × 10^–4^
revM11	5.3 × 10^–6^	1.0 × 10^–6^	N/A	N/A	1.7 × 10^–4^	8.3 × 10^–5^	1.5 × 10^–4^	3.0 × 10^–4^	5.8 × 10^–4^	5.6 × 10^–4^

a The notation is the same as in Table 7; however, cases where the difference between the Gaussian and finite element results is smaller than the accuracy of the finite element results are marked by ∼0.

We observe that the truncation errors are strongly functional dependent, which is not surprising given the analogous FEM data in Table 4 and Table 5. Interestingly, even though un-aug-pc-4 has been optimized for the BLYP functional, it is often not the functional for which the smallest truncation error is observed: the lowest truncation error is often also observed for the PW92 or B3LYP functional. This is of course not surprising, as optimality of the exponents for a given functional does not prevent the truncation error for another functional with the same basis set being smaller.

In the case of the AHGBS-9 basis set, we observe that the smallest truncation errors are achieved for all atoms in case of HF calculations. This can likely be attributed to the quadratic character of the HF energy functional compared to the more complicated mathematical form of DFAs.

#### 4.2.1 Examination into Differences Between un-aug-pc-4 and AHGBS-9

AHGBS-9 generally yields smaller basis set truncation errors than un-aug-pc-4. This means AHGBS-9 is a better basis set, which is not surprising given its large size: the basis sets of ref (39) were designed for high-accuracy calculations on small systems.

Examining the truncation errors further, we observe that AHGBS-9 yields a lower energy than un-aug-pc-4 in 191 calculations. However, we also observe that the reverse is true in 12 calculations: un-aug-pc-4 yields lower energies than AHGBS-9 in the PBE, BLYP, B3LYP, r^2^SCAN01, TASKCC, and ωB97X-noV calculations on Li, and the TPSS, TASKCC, B97M-noV, ωB97M-noV, revM06-L, and M06-SX calculations on Be. The observed issues therefore only affect Li and Be, for which AHGBS-9 reproduces a lower total energy than un-aug-pc-4 in 16 calculations, and a higher one in 12.

In the former case, the largest decreases in total energy going from un-aug-pc-4 to AHGBS-9 are 15 *μE*_*h*_ for Be with the MN15 functional, and 4.9 *μE*_*h*_ for Li with the revTPSS functional. In the latter case, the largest decreases in total energy from AHGBS-9 to un-aug-pc-4 are 94 *μE*_*h*_ for Li with the TASKCC functional, and 57 *μE*_*h*_ for Be with the ωB97M-noV functional.

We shall investigate these discrepancies in the results further by examining the basis sets in detail. The examined configurations of Li and Be only have *s* electrons, and therefore examining the issue reduces to examining the *s* basis functions. Examining the *s* exponents, we see that they span the range 5.93 × 10^–3^*a*_0_^–2^ to 7.07 × 10^4^*a*_0_^–2^ in un-aug-pc-4 for Li, while the corresponding AHGBS-9 basis spans the range 7.05 × 10^–3^*a*_0_^–2^ to 6.85 × 10^5^*a*_0_^–2^, suggesting that AHGBS-9 may not have sufficiently diffuse exponents in the case of Li. However, for Be we observe exponents in the range 1.11 × 10^–2^*a*_0_^–2^ to 1.39 × 10^5^*a*_0_^–2^ in un-aug-pc-4, while in AHGBS-9 the range is 6.51 × 10^–3^*a*_0_^–2^ to 1.22 × 10^6^*a*_0_^–2^, which thus fully covers the range of exponents included in un-aug-pc-4 and suggests that the issue could be a lack of completeness within this range of exponents.

**Figure 1:**
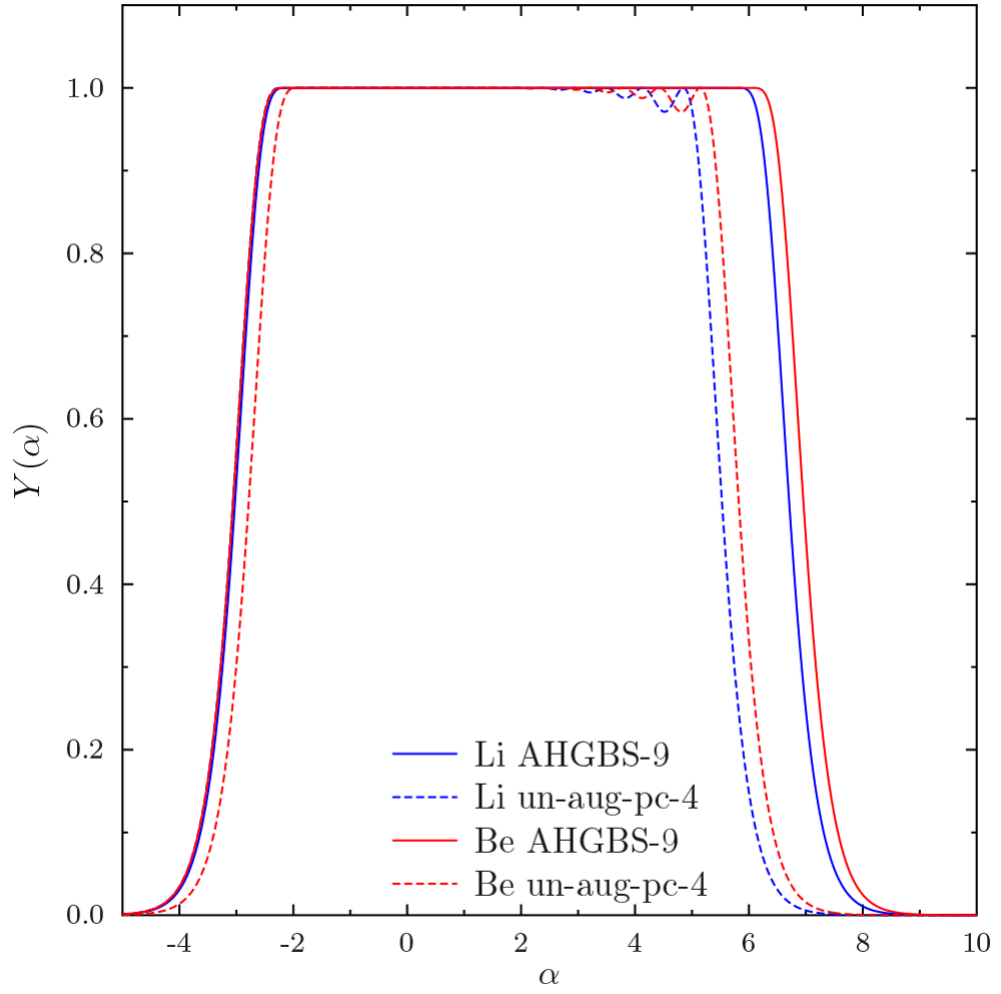
Completeness profile *Y*(α) for the *s* exponents for Li and Be in un-aug-pc-4 and AHGBS-9 basis sets.

The completeness profiles^106^
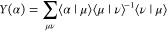
37where α is a test function, μ and ν are atomic basis functions and ⟨μ|ν⟩^–1^ denotes the μ, ν element of the inverse overlap matrix offer a visual tool to inspect the completeness of the studied basis sets. Gaussian functions with exponents α can be expanded exactly in the basis if *Y*(α) = 1, while functions that are orthogonal to the basis set have *Y*(α) = 0. The completeness profiles for the studied *s* functions in the Li and Be basis sets are shown in Figure 1.

**Figure 2:**
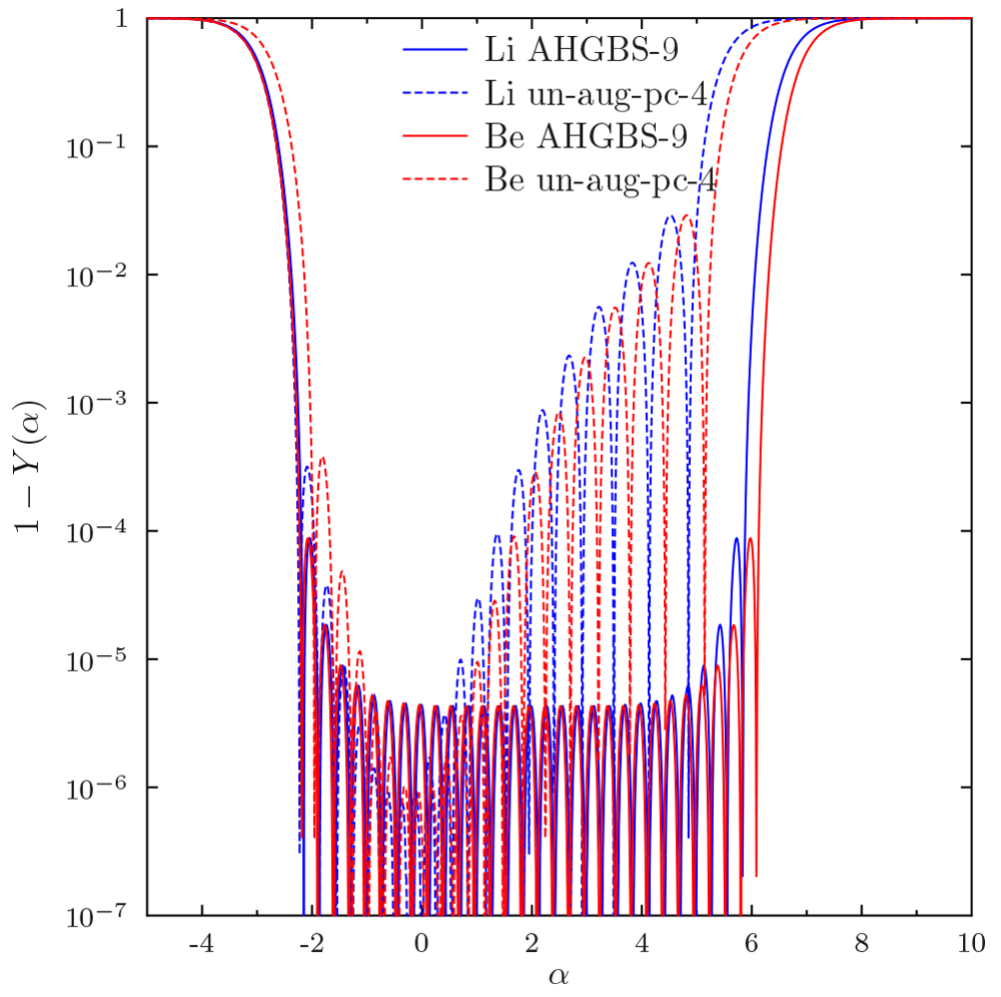
Complementary completeness profile 1 – *Y*(α) for the *s* exponents for Li and Be in un-aug-pc-4 and AHGBS-9 basis sets. Note use of a logarithmic *y* axis.

Figure 1 features oscillations in the profile of un-aug-pc-4 for large exponents α, while the profile for the large AHGBS-9 basis set is flat. These features are even clearer when examining the difference 1 – *Y*(α) in Figure 2: the AHGBS-9 basis appears considerably more flexible than un-aug-pc-4 in the case of Be in the same range of exponents.

We can thus summarize that a more complete Gaussian basis set may reproduce a higher energy than a smaller, less complete Gaussian basis set, depending on the functional. This indicates that the basis set truncation errors observed for various combinations of Gaussian basis sets and density functionals may have nontrivial dependence on the actual exponent values. The dependence of the optimal exponents on the DFA is caused by the differences in the optimal radial orbitals of various functionals. Our data in Tables 7 and 8 demonstrate that the use of a fixed Gaussian basis set can introduce functional dependent errors in total energies in the range of tens to hundreds of microhartree.

These findings again underline the importance of the present contribution in introducing fully numerical methods for the reliable determination of CBS limit energies. The issues discovered with the functional dependence of the exponents also underline another part of our discussion. For example, in the case of Be, using a converged PBE density to start a r^2^SCAN calculation shows that the PBE orbitals yield a total energy which is 0.957 m*E*_*h*_ higher than that for the converged r^2^SCAN orbitals. As we have discussed in ref (12), NAOs are analogous to contracted basis sets, and this error is nothing but the contraction error made when using a minimal NAO basis for the PBE functional in a r^2^SCAN calculation. Although additional basis functions to allow breathing and polarization will allow for energy lowerings, the error of the minimal basis will reintroduce BSSE in calculations. We again underline that NAOs should be formed with the same DFA used in a polyatomic calculation to eliminate errors arising from differences in the optimal form of the radial orbitals.

### 4.3 Representative Timings

Exemplifying the discussion of ref (107) on what kinds of science can be done on today’s commodity hardware with free and open source software, the rapidity of the present implementation in HelFEM is demonstrated by calculations on the author’s laptop running an Intel Core i5–1235U processor. For this demonstration, we choose the Be, Ar, and Xe atoms, and compare the present symmetry aware implementation with the general implementation of ref (37). As methods, we pick HF, PW92, PBE, B3LYP, TPSS, and r^2^SCAN from our previously used selection.

The resulting energies and timings are shown in Table 9. As HelFEM is a new project, the code has not been heavily optimized. Although many things could be done to optimize its performance, the code is usable in present form and fast enough to pursue investigations into the numerical stability of density functional approximations, for instance.

**Table 9:** Timings in Seconds for Various Calculations on the Be, Ar, and Xe Atoms^a^

(a) Be, 15 radial elements					
Method	*E*_gen_	*t*_gen_	*E*_sym_	*t*_sym_	Speedup
HF	–14.5730232	0.4	–14.5730232	0.3	1.5
PW92	–14.4464735	0.6	–14.4464735	0.2	2.6
PBE	–14.6299477	1.8	–14.6299477	0.3	5.7
B3LYP	–14.6733282	1.9	–14.6733282	0.3	6.6
TPSS	–14.6717170	2.6	–14.6717170	0.3	7.5
r^2^SCAN	–14.6490866	2.5	–14.6490866	0.4	6.7

(b) Ar, 15 radial elements					
Method	*E*_gen_	*t*_gen_	*E*_sym_	*t*_sym_	Speedup
HF	–526.8175128	4.0	–526.8175128	0.9	4.5
PW92	–525.9397934	8.2	–525.9397934	0.6	14.7
PBE	–527.3461288	14.2	–527.3461288	0.7	20.1
B3LYP	–527.5678350	12.3	–527.5678350	0.8	15.0
TPSS	–527.5694173	25.0	–527.5694173	0.8	30.5
r^2^SCAN	–527.5177200	24.4	–527.5177200	0.8	30.2

(c) Xe, 25 radial elements					
Method	*E*_gen_	*t*_gen_	*E*_sym_	*t*_sym_	Speedup
HF	–7232.1383639	183.1	–7232.1383639	5.8	31.4
PW92	–7228.8341637	182.3	–7228.8341637	5.1	35.7
PBE	–7234.2332120	296.2	–7234.2332120	7.3	40.6
B3LYP	–7234.8674339	227.9	–7234.8674339	6.1	37.5
TPSS	–7234.4363678	420.1	–7234.4363678	6.9	61.1
r^2^SCAN	–7234.8086847	471.9	–7234.8086847	8.6	55.1

a The first and second columns show the energy reproduced *E*_gen_ and the time *t*_gen_ taken by the general program of ref (37). The third and fourth show the respective values *E*_sym_ and *t*_sym_ for the symmetry-aware program of this work and ref (34). The last column shows the speedup of using symmetry. All energies are in Hartree and times in seconds.

As the timings were obtained on the same machine with largely the same code, they give a good idea of the speedups achieved by the use of symmetry. The most time per iteration in the general program is spent on building the DFA components of the Fock matrix. This is also a part that experiences major speedups due to the use of symmetry to eliminate the angular degrees of freedom, as the quadrature over the solid angle is not needed, nor is the handling and pointwise evaluation of the spherical harmonics. The speedups are larger for DFT than for HF, and increase going from LDAs to GGAs to meta-GGAs.

Although obtaining results for heavy atoms with the general program may take up to tens of minutes, employing symmetry allows obtaining CBS limit results on commodity hardware in a matter of seconds.

## 5 Summary and Discussion

We have presented the formalism necessary to implement meta-GGA functionals in atomic calculations within the finite element method, and implemented it in the free and open source HelFEM program. Furnished with the new implementation, we carried out a large number of calculations with 31 density functionals on the 10 closed-shell or half-closed-shell atoms from H to Ar to determine total energies converged to within 0.1 *μE*_*h*_ with respect to all parameters controlling the calculation: the radial basis set, the quadrature scheme, as well as the density threshold in the density functionals’ implementation in Libxc.^35^ Excluding the nonconverging calculations, we found that a density screening threshold of 10^–11^*a*_0_^–3^ was able to reproduce total energies converged to 0.1 *μE*_*h*_ for all studied functionals.

Ill behavior was observed in several density functionals. The Li and Na atoms proved to be the hardest systems in this study, which we attribute to their extended electronic structure. Pathological behavior was discussed for several functionals for the Li and Na atoms, where the diagonalization of a good initial guess results in thousand-hartree increases of the total energy. This points to issues with large derivatives, which were not examined in our recent study on the numerical behavior of density functionals,^36^ and whose study was one of the central motivations of this work, as fully numerical calculations are stringent tests of density functionals’ behavior.

Equipped with the fully numerical CBS limit energies, we proceeded to study basis set truncation errors in the AHGBS-9^39^ and aug-pc-4^50−55^ basis sets in fully uncontracted form (un-aug-pc-4). The truncation errors were found to be strongly dependent on the functional. Although AHGBS-9 is designed for benchmark studies and is thereby much larger than un-aug-pc-4, we found that un-aug-pc-4 afforded a lower total energy than AHGBS-9 in 12 out of 28 calculations on Li and Be. (For all other systems, AHGBS-9 yielded systematically lower total energies.) Even though un-aug-pc-4 was found to have a more diffuse exponent than AHGBS-9 for Li, in the case of Be the un-aug-pc-4 exponents were found to be included in the range of the exponents for AHGBS-9 and completeness profiles confirmed that AHGBS-9 is a more complete basis set. We therefore concluded that the use of fixed Gaussian exponents can introduce functional dependent errors in the range of tens to hundreds of microhartrees.

Our results underline the importance of fully numerical studies of novel density functionals. The timings presented in this work demonstrate that with the use of symmetry, functionals can be swiftly characterized by a fully numerical calculation. Furthermore, our implementation is open source and is freely available online for anyone for any purpose.

This study is the cornerstone on the road to employing modern finite element techniques for molecular calculations with NAOs. We hope to pursue along the path marked in ref (12) by introducing open source software for NAO calculations in upcoming work. However, as most algorithms required by a NAO program can be formulated independently of other technical choices made in the implementation, our plan is to pursue a modular approach. As we have recently reviewed in ref (107), standard, reusable open source libraries like Libxc^35^ promote peer review, the free exchange of ideas, and maintainability of software, and we are convinced that such libraries merit more attention.
